# UKMenCar4: A cross-sectional survey of asymptomatic meningococcal carriage amongst UK adolescents at a period of low invasive meningococcal disease incidence

**DOI:** 10.12688/wellcomeopenres.15362.2

**Published:** 2019-10-28

**Authors:** Holly B. Bratcher, Charlene M. C. Rodrigues, Adam Finn, Mandy Wootton, J. Claire Cameron, Andrew Smith, Paul Heath, Shamez Ladhani, Matthew D. Snape, Andrew J. Pollard, Richard Cunningham, Raymond Borrow, Caroline Trotter, Stephen J. Gray, Martin C. J. Maiden, Jenny M. MacLennan

**Affiliations:** 1 Peter Medawar Building for Pathogen Research, Department of Zoology, University of Oxford, Oxford, OX1 3SY, UK; 2 School of Cellular and Molecular Medicine, University of Bristol, Bristol, BS2 8AE, UK; 3 Division of Public Health Wales, Cardiff, CF10 3NW, UK; 4 NHS National Services Scotland, Health Protection Scotland, Glasgow, G2 6QE, UK; 5 University of Glasgow Dental School, Glasgow, G2 3JZ, UK; 6 Scottish Microbiology Reference Laboratory, NHS Greater Glasgow & Clyde, Glasgow, G2 6QE, UK; 7 Paediatric Infectious Diseases Research Group, St George’s, University of London, London, SW17 0QT, UK; 8 Immunisation Department, Public Health England, London, UK; 9 Oxford Vaccine Group, Department of Paediatrics, University of Oxford, Oxford Biomedical Research Centre, Oxford, OX3 7LE, UK; 10 Microbiology Department, University Hospitals Plymouth NHS Trust, Plymouth, PL6 8DH, UK; 11 Meningococcal Reference Unit, Public Health England, Manchester Royal Infirmary, Manchester, M13 9WL, UK; 12 Disease Dynamics Unit, Department of Veterinary Medicine, University of Cambridge, Cambridge, CB3 0ES, UK

**Keywords:** Neisseria meningitidis, UKMenCar, adolescent, meningococcal carriage, population immunity, population genomics

## Abstract

Carriage of *Neisseria meningitidis*, the meningococcus, is a prerequisite for invasive meningococcal disease (IMD), a potentially devastating infection that disproportionately afflicts infants and children. Humans are the sole known reservoir for the meningococcus, and it is carried asymptomatically in the nasopharynx of ~10% of the population. Rates of carriage are dependent on age of the host and social and behavioural factors. In the UK, meningococcal carriage has been studied through large, multi-centre carriage surveys of adolescents in 1999, 2000, and 2001, demonstrating carriage can be affected by immunisation with the capsular group C meningococcal conjugate vaccine, inducing population immunity against carriage. Fifteen years after these surveys were carried out, invasive meningococcal disease incidence had declined from a peak in 1999.  The UKMenCar4 study was conducted in 2014/15 to investigate rates of carriage amongst the adolescent population during a period of low disease incidence. The protocols and methodology used to perform UKMenCar4, a large carriage survey, are described here.

## Introduction

Asymptomatic oropharyngeal carriage of the Gram negative diplococcus *Neisseria meningitidis* occurs at a variable rate, with a range of approximately 2% to 30%, dependent on age and exposure to risk factors ^1^. Humans are the sole known reservoir for the meningococcus, and as such it is an obligate human commensal and pathogen. Transmission of meningococci occurs by droplet spread through close contact with an infected individual. Of those who carry meningococci, a very small number, 1–2 per 100,000 in the UK ^2^, will develop invasive meningococcal disease (IMD) with the bacteria invading systemically through the oropharyngeal epithelium, resulting in septicaemia and/or meningitis.

A meta-analysis of meningococcal carriage in Europe, North America and Australia, where serogroups B and C IMD predominates, demonstrated increasing carriage with age, with low carriage in young children to 23.7% in 19 year olds, subsequently declining in adulthood to 7.8% in 50 year olds ^3^. Risk factors that affect carriage include; living in overcrowded settings; passive and active smoking; intimate contact (e.g. kissing); frequenting pubs or clubs; and intercurrent viral respiratory tract infection ^4– 6^. Carriage rates are dynamic and have been observed to rise in UK students starting university, from 6.9% on the first day of university term to 23.1% by day four ^7^. Carriage rates as high as 60–70% have been reported amongst military personnel, with disease outbreaks a common occurrence in both these settings ^8, 9^. There is variation in meningococcal carriage and disease epidemiology globally. For example, historically high IMD incidence in the meningitis belt in Africa, led to carriage studies ^10^ performed by the MenAfriCar consortium in association with the introduction of the conjugate polysaccharide A vaccine in 2010. These surveys identified mean carriage prevalence of 4.5%, lower than high IMD incidence, non-African countries, with the highest rates amongst 5–14-year-olds in the belt ^11, 12^. Risk factors in this setting included living in rural communities and the dry seasonal climate ^12^.

The human nasopharynx and oropharynx are important sites of bacterial colonization supporting a complex and changing microbiota. Awareness and knowledge of the complex association of the microbiota is critical to understanding immune response and preserving human health as well as its relationship to invasive infection. For example, the Human Microbiome Project identified Bacteroidetes and ** Proteobacteria as two of the core taxonomic groups within the throat of healthy individuals ^13^. In addition, this study found an inverse relationship exists between the presence of Bacteriodetes and Proteobacteria, which includes the genus *Neisseria*. Studies of meningococcal carriage prevalence have identified an inverse relationship between pathogenic and non-pathogenic *Neisseria* species ^1, 14, 15^. The studies also indicate that host age, as well as a predominant species, influenced host susceptibility to colonisation and invasion by the pathogenic species, *Neisseria meningitidis* ^16, 17^. 

A relationship between asymptomatic carriage and IMD outbreaks has been recognized since pioneering work conducted in the UK in the First World War ^18^, where increased meningococcal carriage as a consequence of overcrowding and social interactions was found to result in increased rates of IMD ^3, 19^. There is genotypic and phenotypic diversity amongst the meningococci carried in the oropharynx and certain meningococci are inherently more invasive than others. Many genetic constituents underlay the phenotypic expression of a meningococcus; for example, the function of capsular expression and other virulence factors associated with particular genetic lineages, can be defined by multilocus sequence typing (MLST) as a clonal complex (cc) ^12, 20– 22^. In response to an increase in IMD with associated high mortality due to a serogroup C, hyperinvasive clonal complex ST-11 (C:cc11) the capsular group C meningococcal conjugate vaccine (MCC) was introduced into the UK immunisation schedule in November 1999, with a catch up campaign for all <18 year olds ^23^. Three large multi-centre carriage surveys conducted through the UK Meningococcal Carriage (UKMenCar) consortium, UKMenCar1-3, were carried out in 1999, 2000, and 2001. These surveys assessed oropharyngeal carriage of meningococci and collected risk factor data in over 45,000 adolescents in the UK; and demonstrated that the population immunity induced by the MCC vaccine programme was due to the reduction in carriage of the specific hypervirulent strain C:cc11 ^24, 25^.

In the UK, the greatest burden of endemic IMD has been due to serogroup B since the 1980s ^26^. Unlike the hyperendemic periods, which usually represent a clonal expansion of a novel strain, endemic disease is caused by multiple different genetic lineages of meningococci, which are influenced by secular changes ^22^. There has been a progressive fall in overall serogroup B IMD in the UK since 2000–2001, when in England 1614/2343 (69%) cases were due to serogroup B compared with 418/724 (58%) in 2014–2015, predominantly affecting infants and children under five ^26, 27^. The reasons for this decline in serogroup B IMD were unclear, in the absence of any national vaccination programme targeting capsular group B meningococci. Given the changing epidemiology since 1999, this UKMenCar4 study was performed in 2014–2015 to undertake a structured investigation of the difference in adolescent meningococcal carriage between periods of high (1999–2001) and low (2014–2015) IMD incidence using both the phenotypic and genotypic information.

## Protocol

### Study aims

The aims of the UKMenCar4 study were to: i) characterise a representative collection of meningococcal carriage isolates from UK adolescents during a period of low IMD incidence; ii) identify risk factors associated with meningococcal carriage; and iii) to study the relationship between meningococcal carriage and disease during periods of high and low IMD incidence.

### Study objectives

The specific objective was to characterise the *Neisseria* species obtained as part of the UKMenCar4 study, which sampled 21,873 adolescents aged 15–19 years attending educational establishments in eleven sampling sites around the UK between September 2014 and March 2015. These isolates were characterized phenotypically and genotypically in order to:

(i) identify the *Neisseria* species present, determining carriage rates for *N. meningitidis* (the meningococcus) and other *Neisseria* species;
(ii) establish the meningococcal lineages present using whole genome sequence data;
(iii) quantify the meningococcal capsule type prevalence and rates of expression;
(iv) make the data publicly-available via the https://pubmlst.org/neisseria/ website ^28^.

### Study design

The UKMenCar4 study was a cross-sectional survey of meningococcal carriage conducted from September 2014 to March 2015. Sample collection was funded by the Wellcome Trust (087622/Z/08/Z) and a Wellcome Trust Institutional Strategic Support Fund and molecular characterization of isolates was funded by the UK Department of Health and National Institute for Health Research (NIHR) Policy Research Programme (PR-ST-0915-10015). This study used the same approach and sampling methodology as UKMenCar1-3 studies, conducted from 1999–2001, in order to ensure comparability ^20, 24, 25^. Previous carriage studies conducted during UKMenCar1-3 had estimated carriage in the 15–19-year-old age group at 16.7–18.7% ^25^. A sample size of 18,000 participants was calculated to provide equivalent numbers of meningococci to UKMenCar1-3 studies, assuming a carriage prevalence of 17% ^25^. It was anticipated that around 3000 meningococcal isolates would be collected, allowing the prevalence of rare strains to be determined with good precision, e.g. variants with 1% prevalence, 95% confidence interval 0.85% to 1.15%. Achieving a very large unbiased cohort size required a multi-centre design, involving centres which had a proven recruitment track record. The sites were chosen to provide broad geographical distribution throughout the United Kingdom and encompassing a wide range of demographics. Schools and colleges were approached directly and asked if they would be willing for students in their institution to take part and pre-recruitment numbers were estimated, and details regarding the study were made available on the school/college website and distributed via newsletter where available. To ensure adequate enrolment two additional centres were recruited through the clinical trials network.

### Study participants

**Figure 1.**
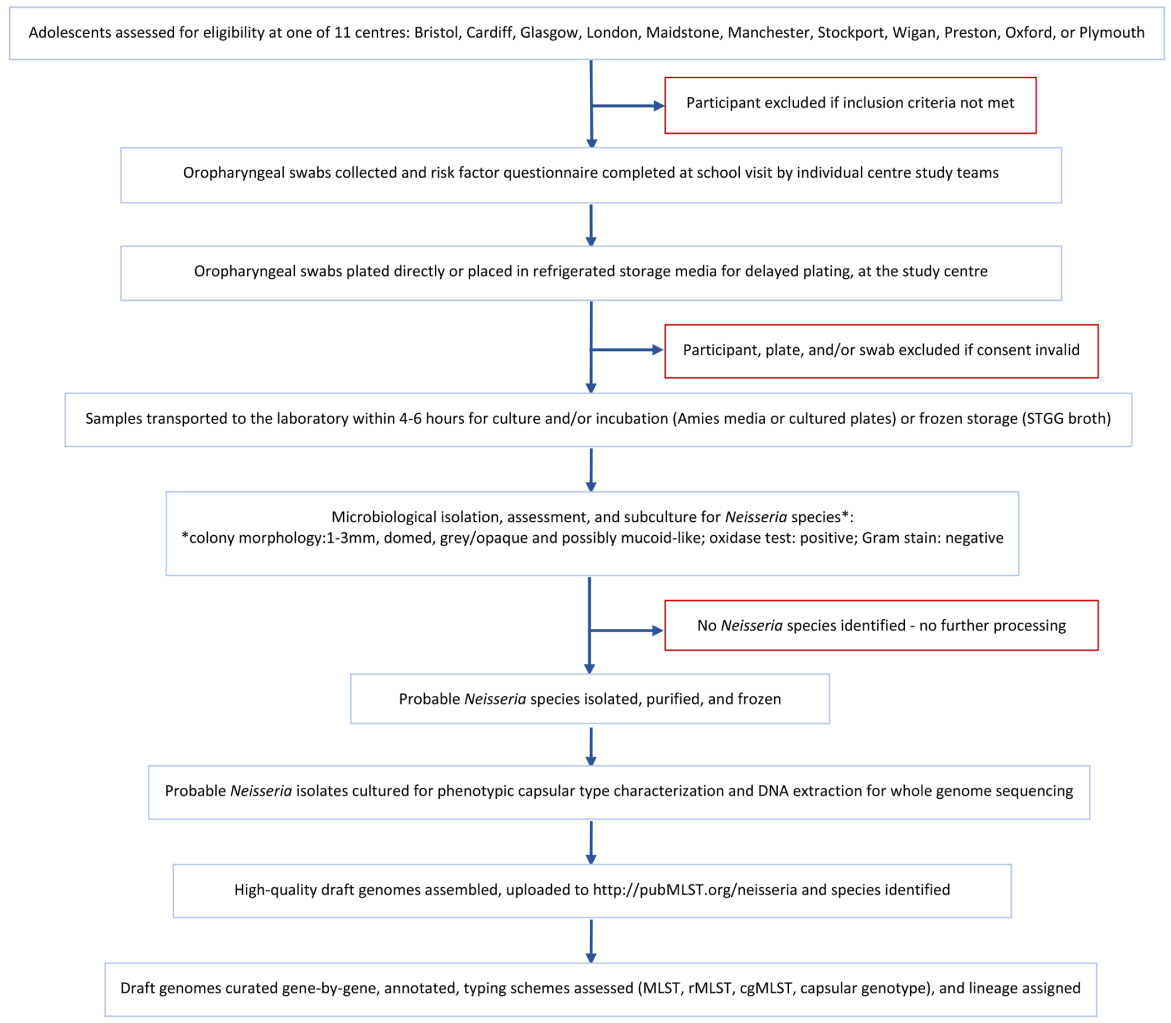
Summary overview of the UKMenCar4 study protocol. A cross-sectional survey of meningococcal carriage, conducted from September 2014 to March 2015.

**Figure 2.**
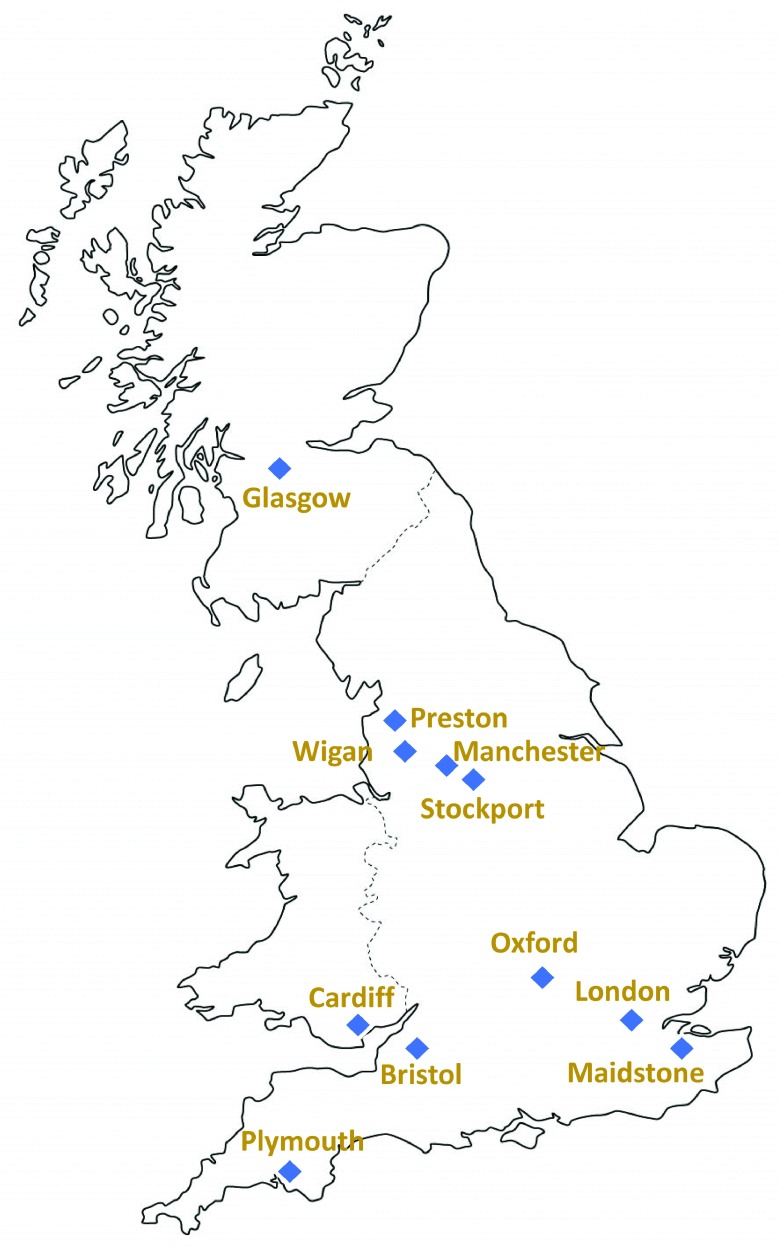
UKMenCar4 study centres, representing England, Scotland and Wales. Sampling the ethnic diversity of UK cities through comprehensive schools, independent schools, and voluntary aided schools, sixth form colleges and technical colleges, mixed and single sex schools, day schools and others with boarding facilities, located in areas with different income bands.

Adolescents and young adults who were 15–19 years old, attending school or college in UK academic years 12 and 13 or equivalent, were invited for oropharyngeal swab collection and completion of a risk factor questionnaire ( *Extended data*, file 1) ^29^ following circulation of study information prior to school visits by study staff ( Figure 1). Students were recruited to take part through local schools/colleges in 11 sampling centres: Bristol (West England); Cardiff (South Wales); Glasgow (Scotland); London; Maidstone (South-East England); Manchester; Stockport; Wigan; and Preston (North-West England); Oxford (South-Central England); and Plymouth (South-West England) ( Figure 2). The full information sheet ( *Extended data*, file 2) ^29^ was provided in advance of the study for parents to discuss with their children and provided the information required for informed consent. Adolescents in many schools/colleges acted as ‘School Ambassadors’, assisting in practical aspects of the study. The study was approved under the NHS Research Ethics Committee reference 14/SC/1163 (Integrated Research Approval System 154609).

### Sample collection

Written, informed consent was obtained by all study participants. After informed consent, individual students were sampled with an oropharyngeal swab, inserted past the uvula and sweeping the back of the throat. The swab was either inoculated directly onto a selective growth media plate (GC agar base containing VCAT (vancomycin, colistin, amphotericin and trimethoprim) and incubated within 4 hours) or placed directly into STGG (skim milk, tryptone, glucose, glycerol) broth for transport to a local laboratory for plating (within 4–6 hours). If swabs collected into STGG broth could not be plated in the laboratory within 4 hours, they were frozen at -80°C, then thawed and cultured as soon as laboratory capacity allowed. Students completed a questionnaire including demographic information and risk factors for meningococcal carriage. All swabs, plates and/or tubes were labelled with a pre-printed unique participant accession number and cross-referenced to the questionnaire and consent form. 

### Microbiological culture

After incubation at 37°C, 5% CO _2_ for 16–24 hours, a representative colony of putative *Neisseria* was sub-cultured onto non-selective Columbia blood agar (CBA) plates to obtain a pure, fresh culture for further characterisation. Following incubation at 37°C, 5% CO _2_ for a further 16–24 hours, pure sub-cultured colony growth was characterised by Gram stain and oxidase reaction. Gram negative, oxidase positive diplococci were stored in duplicate at -80°C in brain heart infusion and 20% glycerol at the processing site or sent to the University of Oxford, UK. A sample of each recovered putative *Neisseria* isolate was sent to the Public Health England Meningococcal Reference Unit (PHE-MRU) in Manchester, UK.

### Phenotypic characterization and DNA extraction

At the PHE-MRU, isolates were revived and phenotypically characterised for capsular types B, C, W, and Y using standard serological techniques ^30^. DNA extraction was performed using the DNA extraction using the Qiagen DNeasy Blood and Tissue Kit (Qiagen, Crawley, UK). A single colony archived culture was used to avoid selection of unrepresentative phase variants. Purified DNA was quantified and sent to the Oxford Genomics Centre (OGC, Wellcome Trust Centre for Human Genetics, University of Oxford, UK) for whole genome sequencing using the Illumina HiSeq platform.

### Whole-genome sequencing and genomic analysis

Short-read sequence data were assembled using Velvet (version 1.2.10) with VelvetOptimiser (version 2.2.4) assembly package with an established pipeline ^31^. The assembled high-quality draft genomes were uploaded onto the pubMLST.org *Neisseria* database, which includes the Meningococcal Research Foundation Meningococcal Genome Library (MRF-MGL) amongst other meningococcal collections ^26^. Corresponding FASTQ files are available from the European Nucleotide Archive (study reference PRJEB14319). Each genome was annotated using *Neisseria* locus tag identifiers for >2,400 individual loci, including loci belonging to the MLST ^22^, ribosomal MLST ^32^, core genome MLST ^31^, capsular genogroup ^33^, and Bexsero® Antigen Sequence Typing (BAST) ^34^ schemes. Using a gene-by-gene analysis approach, isolates that belonged to the genus *Neisseria* and were determined and speciated; each isolate was then characterised by sequence type (ST), cc, genetic lineage, capsular genogroup, and BAST (protein-based vaccine Bexsero® variants) profile.

### Data analysis

Data from the UKMenCar4 survey (2014/15) was analysed alongside results from UKMenCar1-3 surveys (1999, 2000, and 2001) for comparison. The UKMenCar4 dataset was prepared by reconciling the WGS data with participant questionnaires. Samples were excluded if essential questionnaire data were incomplete, or if valid consent was missing. All data and statistical analyses were performed using Intercooled Stata (version 14) ^35^. Initially, individual level risk factors for meningococcal carriage will be analysed in single-variable models by using logistic regression. A multi-level modelling approach will then be used with levels defined as: level 1 individuals, level 2 school, and level 3 centre. Univariable analyses will identify risk factors at each level with p<0.2, for inclusion in a multilevel logistic regression model for a multivariable analysis. We will also compare findings between (a) subjects carrying any meningococcus, (b) those carrying a meningococcus associated with invasive disease and (c) those who are not carrying a meningococcus, to allow any specific risk factors for disease associated meningococcal carriage to be identified. The meningococcal population distribution is described using point prevalence for each serogroup with 95% confidence intervals and case-carrier ratios for different strains will be calculated to look for relative virulence between 1999 and 2014 in the carrier population according to circulating disease types. The meningococcal genomes and associated risk factor data will be publicly-available on https://pubMLST.org/neisseria on final acceptance and publication of results.

### Data storage and dissemination

Each participant was given a single, unique study number, on pre-printed labels, to reduce errors and to facilitate data linkage and samples. The name of participants and corresponding study number are kept on a separate and secure database. Centres used their preferred method of data capture – paper questionnaires or electronic capture. Data was double entered, verified, and stored at the site where collected prior to incorporation into a central database. Subject associated data is stored securely at the study site and only accessible by authorised study personnel.

An annual progress report and an end of study final report were produced and participating schools informed. Study data, isolate characterization, and risk data analysis results will be published in peer reviewed scientific journals and provided in conference presentations. The collected information will also be publically accessible via PubMLST.org in the *Neisseria* database using the project name “UKMenCar4”.

### Equality and diversity

Survey sites included comprehensive, independent, and voluntary-aided schools, sixth form and technical colleges, mixed- and single-sex schools, day schools and others with boarding facilities, and located in areas with different income bands. Participation was voluntary and those willing to take part were random, with the expectation that the cohort was a representative sample of the communities from which they were obtained, with diversity of gender, ethnic, social, educational, and income status. 

### Patient and public involvement in research

Throughout the UKMenCar4 study, the management group engaged with charities (Meningitis Research Foundation and Meningitis Now), patient groups (individuals affected by IMD and their carers), and prospective participants, who were involved in discussions of the study motivation and design. Adolescents aged 15–19 years piloted information sheets and questionnaires with their feedback incorporated, including the addition of a shorter summary information sheet. ‘School Ambassadors’ were involved in ensuring the smooth running of the study on site as well as workshops, laboratory visits, and feedback sessions. Working alongside charities, public awareness information about meningitis was provided including presentations at school assemblies and information cards listing meningitis symptoms and advice. During the collection of UKMenCar4 samples, a three-session course was piloted with a group of 10 year 12 students. The course comprised:

(i) a one-hour lecture: ‘At the back of your throat: meningococcal disease and adolescents’, which described the basic biology of the meningococcus and the importance of transmission and population immunity in prevention of meningococcal disease by immunisation;
(ii) a one-hour computer practical session: ‘How to be a genome detective’, in which the students were able to use the web-based tools in the MRF-MGL to investigate the epidemiology of meningococcal disease in the UK;
(iii) a one-hour tutorial on the implementation of meningococcal vaccines. Working in groups of three with a tutor, students were provided with various documents in advance and were asked to argue from the following perspectives: a patient’s parent; a paediatrician; a government official; a vaccine manufacturer. The tutorials explored the basis of decisions on immunisation and the different conclusions that different individuals might make from the same information.

## Discussion

Humans are the sole known reservoir for the meningococcus and, as such, carriage surveys can provide information about the human reservoir and transmission dynamics that have implications for disease epidemiology. Studying the physiological circumstances that promote bacterial invasion rather than colonization is difficult as human challenge models are not appropriate, but both laboratory ^36^ and mouse ^37, 38^ models have been developed to address these research questions *in vitro*. Despite this, real life population dynamics are relatively easy to measure and monitor through large carriage surveys, though to obtain a sample representative of the whole population, studies need to be of sufficient magnitude and well-resourced.

Information collected in the UKMenCar1-3 studies demonstrated that the MCC vaccine induced population immunity by specifically reducing carriage of C:cc11, and that the high rates of transmission in this age group was a consequence of social behaviour, especially smoking, kissing, and attendance at pubs and clubs ^6^. In 2014, the UKMenCar consortium was reconvened in order to conduct a fourth multi-centre carriage survey (UKMenCar4), aiming to provide a large-scale survey of meningococcal carriage at a time of low IMD incidence. The UKMenCar4 study provides an extensive resource for addressing meningococcal epidemiology. Understanding the transmission dynamics and changes in carriage over time, will help to explain the decline in IMD in the UK from 1999–2019. Characterisation of the *Neisseria* species from this study will provide a definitive molecular description of the prevalence and types of meningococci carried by UK adolescents in 2014/15. In this study an emphasis was placed on maintaining consistency of sampling and analysis. Therefore, UKMenCar4 methodology was conducted as with previous UKMenCar studies to ensure longitudinal comparability. Meningococcal carriage was defined by the presence of at least one colony of typical *Neisseria* species morphology on an isolation plate, with reliance on microbiological identification. Further biochemical tests were used to confirm the Gram negative, oxidase positive diplococci and WGS to determine *Neisseria* species.

Conventional microbiological approaches to identifying the oropharyngeal microbiota has revealed *Neisseria* to be abundant ^39, 40^. Standardized collection and laboratory identification methods that are robust, scalable, and cost-effective for large studies contribute to the overall interpretation and understanding of *Neisseria* epidemiology and its diversity. The UKMenCar4 study aimed to collect, identify, and genome sequence all recovered *Neisseria* species using methods that allowed comparison with the UKMenCar 1-3 surveys. Literature describing the methods of sampling, identifying, and measuring meningococcal carriage is abundant; however, at the time of writing there was no universally agreed method for carriage studies within the academic community, with choice of protocol based on specific study questions and aims. Following on from the UKMenCar1-3 surveys (1999–2001), large carriage studies were undertaken by the MenAfriCar consortium prior to the introduction of the conjugate polysaccharide A vaccine in 2010 for use in the African meningitis belt. Oropharyngeal as opposed to nasopharyngeal swabbing was performed in 19 of 20 carriage surveys, largely swabbing the posterior pharyngeal wall ^11^. Comparison of swabbing sites between the posterior pharynx behind the uvula and the posterior pharynx behind the uvula and one tonsil, in a direct comparison in Mali, identified that carriage where only one site is swabbed may underestimate carriage rates ^41^. Alternative samples, such as saliva, are rapid, inexpensive, and non-invasive but there are conflicting reports on their effectiveness for detecting carriage of *Neisseria* ^42– 44^. The UKMenCar surveys sought to optimize the site of swabbing by taking a sweep of the posterior pharynx from one tonsil to the other.

Once swab samples are obtained, different methods can be used for microbiological identification of meningococci. Some studies have indicated that PCR methods are an alternative means of identifying *Neisseria* from clinical samples and have been shown to increase reported cases of IMD ^45^. A large multi-centre study in South Australia, studying the impact of 4CMenB vaccine Bexsero® on adolescent carriage, undertook DNA extraction from swabs and real-time PCR screening for *porA* for identification of meningococci and capsular group, followed by selective culture of PCR positive swabs. This has the benefit of reducing the microbiological time, labour and expense of culturing all oropharyngeal swabs and increasing sensitivity to low colonization density. However, the disadvantages include the inability to identify other *Neisseria* species that lack *porA*, or other targets ( *ctrA*, *siaD*), such as *Neisseria lactamica*, or the impact of phase variation on capsular expression ^11, 46^. Both these variables contribute to the understanding of the oropharyngeal microbiota and the complex interactions and relationships between the different *Neisseria* species that occupy this niche at different ages ^17^, with implications for carriage and, therefore, disease due to *N. meningitidis.* A human challenge model of *N. lactamica* inoculation demonstrated inhibition of *N. meningitidis* colonization ^16^, with potential implications for prevention of IMD, though the nature of the intra-species relationships within the microbiota remain unclear. Comprehensive studies of the interactions within the oropharyngeal microbiota will increase our ability to define risk factors that predispose to carriage and progression to invasive disease, and develop preventative strategies.

Through WGS analysis of carriage isolates, it is possible to determine the antigenic variation of protein antigens present in the protein-based vaccines. Hence, the UKMenCar4 study also serves as a baseline to analyse further changes in meningococcal carriage in the UK after the introduction of 4CMenB vaccine into the infant immunization schedule in September 2015. Lessons learned from UKMenCar1-4 methodology have been important for the efficient design of trials in South Australia ^24^ and the UK to evaluate the effect of protein-based vaccines on meningococcal carriage, especially pathogenic meningococci and genogroup B isolates. Such studies can only be powered adequately with knowledge of the background prevalence of carriage of different types of meningococci, information provided by UKMenCar4.

*Neisseria* are a highly abundant component of the human pharyngeal microbiota and it remains a challenge to understand how carried populations contribute to human health and what impact carriage has on colonization and prevention of disease caused by the hyperinvasive meningococcal lineages in the vast majority of people. Meningococcal carriage studies have played an important role in developing our understanding of the population biology, genotypic and phenotypic epidemiology, as well as the risk factors and pathogenesis of IMD. Given the epidemiology of IMD is dynamic, through secular changes ^47^ or vaccine interventions ^25^, ongoing surveillance of both carriage and disease is crucial for this infectious disease that contributes to a significant proportion of morbidity and mortality.

## Data availability

### Underlying data

No underlying data are associated with this article.

### Extended data

Figshare: UKMenCar4 survey protocol forms. https://doi.org/10.6084/m9.figshare.8952986.v1 ^29^.

This project contains the following extended data:

- extended_data_dile_1.pdf. (Study Questionnaire, used to obtain information from study participants regarding social demographics and risk factors known to be associated with carriage of the meningococcus.)
- extended_data_file_2.pdf. (Study information sheet describing the survey and the type of studies the data will be supporting. The sheet was given to students prior to their enrolment, to provide ample time to consider the information, and the opportunity to question the Investigator, their GP or other independent parties to decide whether they will participate in the study. Sections highlighted in yellow were adapted to reflect the details of the individual Centres.)

Data are available under the terms of the Creative Commons Attribution 4.0 International license (CC-BY 4.0).

**Extended data file 1. Study Questionnaire.** Form used to obtain information from study participants regarding social demographics and risk factors known to be associated with carriage of the meningococcus.

**Extended data file 2. Study Information Sheet.** An information sheet describing the survey and the type of studies the data will be supporting. The sheet was given to students prior to their enrolment, to provide ample time to consider the information, and the opportunity to question the Investigator, their GP or other independent parties to decide whether they will participate in the study. Sections highlighted in yellow were adapted to reflect the details of the individual Centres.
