# Supplementary material for: UKMenCar4: A cross-sectional survey of asymptomatic meningococcal carriage amongst UK adolescents at a period of low invasive meningococcal disease incidence
Source: Wellcome Open Res. 2019 Oct 28;4:118. Originally published 2019 Aug 6. [Version 2] doi: 10.12688/wellcomeopenres.15362.2 (PMC6749934; doi:10.12688/wellcomeopenres.15362.2)
Supplement: Peer review history

# Peer review: Reviewer response for version 1

**DOI:** 10.21956/wellcomeopenres.16781.r36292

**Published:** 2019-09-17

**Authors:** Annaliesa S. Anderson

**Affiliations:** 1 Vaccine Research and Development, Pfizer, Pearl River, NY, USA

Invasive meningococcal disease (IMD), caused by *Neisseria meningitidis*, though rare has a rapid onset and is associated with severe outcomes. Vaccines are now available for the five serogroups that cause the majority of disease. These vaccines have mostly been licensed by demonstrating that they induce antibodies that can kill their target pathogen in serum bactericidal assays. This approach has resulted in a reduction of disease rates for *N. meningitidis* serogroup C (MnC) after the introduction and mass vaccination of children in the UK from 1.8 cases per 100000, prior to introducing the vaccine in 2000, to 0.08 cases per 100000 in 2015/16 ^1^. The primary risk factor for IMD, is coming into contact with the pathogen, specifically through the oropharyngeal route. Carriage studies conducted after the implementation of the MnC conjugate vaccine demonstrated that it not only reduced disease incident rates, but also reduced the carriage of MnC strains in the population ^2^^,^^3^. Thus the vaccine provided direct protection from disease by making antibodies that killed MnC and indirect protection by inducing herd protection by preventing acquisition of the pathogen. Carriage studies, such as UKMenCar4 are an incredibly important approach to indirectly measure the potential effectiveness of vaccines through herd immunity. They can also provide an insight into population dynamics of the pathogen in different environments as a way to better understand additional risk factors for IMD and to identify the optimum ages to vaccinate children prior to them becoming at risk.

 UKMenCar4 is a cross-sectional survey of 21,873 UK adolescents aged between 15 and 19 years. It was conducted as a follow-on study of those conducted 19 years previously when meningococcal carriage rates were approximately 17% in this age group; and disease rates were 9.28 per 100000 I 15-17 year olds ^4^. A major objective for this study is to assess what the carriage dynamics are in a period of low IMD incidence. The submitted protocol provides the justification for the study- size, population, collection methods, data analysis and meningococcal isolate characterization. The protocol also provides information on how the different study sites use student advocates to disseminate information to the student bodies.

 Bratcher *et al *discuss how some of the protocol choices were made. A major consideration is the approach that is used to define carriage. *N. menigitidis* strains are fastidious which has led to the practice of plating out the subjects swabs as soon as they are taken in the field as opposed to transporting them to a laboratory prior to doing the isolation work. In contrast with the advent of sensitive molecular diagnostics many groups have assessed whether the culture approach can be negated by directly analyzing the swab for DNA. Jones *et al* ^5^ conducted a head to head analysis of carriage and disease isolates using molecular approaches and showed that while the invasive disease isolates (99%) could be readily characterized by PCR genogrouping approaches, only 43% of isolates could be grouped by this approach. Sequencing of these isolates revealed that small changes in the capsular operon was the culprit for this lack of sensitivity, highlighting the importance of collecting the isolate so that more definitive strain characterization can be conducted. This still leaves the question of what may be being missed due to the potential for isolation approaches to be less sensitive than molecular methods. A series of studies in 10 – 25 year old’s (approximately 4000) were recently conducted in the UK ^6^, Canada ^7^ and the Netherlands ^8^ to specifically address this question. The overall meningococcal carriage rates were similar for both methods. In contrast there was a trend for *N. menigitidis* serogroup B rates to be lower in the UK and Canada for the cultured samples compared to the samples that were not cultured. These studies were designed to pinpoint at what age subjects start to carry meningococcal strains and thus looked at a wider age range than the described UKMenCar4 study. In Canada carriage rates jumped from approximately 8% to 25% as subjects transitioned to a University setting. In the Netherlands carriage rates started to rise in 14- 18 year olds and averaged around 20% for older subjects. In the UK there was on incremental increase of approximately 10% between the 15-16, 17-18 and 19-25 year old age groups from approximately 20% to 40%. The younger age groups had much lower carriage rates; suggesting that from a vaccination perspective it would be important to ensure that children are vaccinated to ensure that they produce an optimal response prior to being exposed to the pathogen if vaccination is indicated. These studies also highlight that although smaller than UKMenCar4, carriage rates may differ from region to region, which should also be taken into consideration when implementing meningococcal vaccine schedules.

 In summary the UKMenCar4 protocol will be sufficient to estimate UK carriage rates in this population. It will not have the advantage to determine what the trends are in the aligning age groups but will provide a useful baseline is meningococcal vaccine schedules are expanded to adolescents in the UK.

I confirm that I have read this submission and believe that I have an appropriate level of expertise to confirm that it is of an acceptable scientific standard.

## References

[1] : Serogroup C Neisseria meningitidis disease epidemiology, seroprevalence, vaccine effectiveness and waning immunity, England, 1998/99 to 2015/16. *Euro Surveill*.2019;24(1) : 10.2807/1560-7917.ES.2019.24.1.1700818 10.2807/1560-7917.ES.2019.24.1.1700818 30621818PMC6325668

[2] : Impact of meningococcal serogroup C conjugate vaccines on carriage and herd immunity. *J Infect Dis*.2008;197(5) : 10.1086/527401 737-43 10.1086/527401 18271745PMC6767871

[3] : Herd immunity from meningococcal serogroup C conjugate vaccination in England: database analysis. *BMJ*.2003;326(7385) : 10.1136/bmj.326.7385.365 365-6 10.1136/bmj.326.7385.365 12586669PMC148893

[4] : Impact of meningococcal C conjugate vaccine in the UK. *J Med Microbiol*.2002;51(9) : 10.1099/0022-1317-51-9-717 717-22 10.1099/0022-1317-51-9-717 12358061

[5] : Comparison of Phenotypic and Genotypic Approaches to Capsule Typing of Neisseria meningitidis by Use of Invasive and Carriage Isolate Collections. *J Clin Microbiol*.2016;54(1) : 10.1128/JCM.01447-15 25-34 10.1128/JCM.01447-15 26311858PMC4702718

[6] : Meningococcal carriage in adolescents in the United Kingdom to inform timing of an adolescent vaccination strategy. *J Infect*.2015;71(1) : 10.1016/j.jinf.2015.02.006 43-52 10.1016/j.jinf.2015.02.006 25709085

[7] : A Longitudinal Epidemiology Study of Meningococcal Carriage in Students 13 to 25 Years Old in Quebec. *mSphere*.2018;3(6) : 10.1128/mSphere.00427-18 10.1128/mSphere.00427-18 PMC628200830518675

[8] : Meningococcal carriage in Dutch adolescents and young adults; a cross-sectional and longitudinal cohort study. *Clin Microbiol Infect*.2017;23(8) : 10.1016/j.cmi.2017.02.008 573.e1-573.e7 10.1016/j.cmi.2017.02.008 28192234

# Peer review: Reviewer response for version 1

**DOI:** 10.21956/wellcomeopenres.16781.r36276

**Published:** 2019-09-06

**Authors:** Derek J. Fairley

**Affiliations:** 1 Belfast HSC Trust, Belfast, UK; 2 Queen's University Belfast, Belfast, UK

Meningococcal carriage, especially among adolescents, is a critical part of the natural history of meningococcal disease. Adolescents represent the main reservoir of this pathogen, and the changing epidemiology of this disease in the UK and elsewhere is driven by changing patterns of carriage. In addition, the longer term effects of vaccination on rates and patterns of meningococcal carriage and disease are rather uncertain. Comprehensive carriage studies are essential to improve our understanding of both pathogenic and non-pathogenic *Neisseria *species, and as surveillance tools.

 This protocol describes such a study, the fourth in a series conducted by the UK Meningococcal Carriage consortium, in England and Wales.

 Significantly, this carriage study also serves as a baseline immediately prior to the introduction of routine infact MenB vaccination in the UK in 2015.

 As for previous UKMenCar studies, the study aimed to recruit a large and unbiased cohort of adolescents through a multi-centre cross-sectional cohort design. The study team are internationally recognised experts in this field, and the methods were chosen to ensure compatibility with previous UKMenCar studies.

 I would encourage future studies of this type to consider molecular testing of naso/pharyngeal swab specimens in addition to culture to identify carriers.

 I was slightly disappointed to see the oft-cited average carriage rate of ~10% appear prominently in the Abstract (albeit with the caveat that rates "are dependent on age of the host and social and behavioural factors"). The authors also cite (from a 2010 meta-analysis) a figure of 4.5% carriage in infants. That analysis did not assume zero carriage at age zero, and the fitted data indicated 4.5% meningococcal carriage at birth - a value I feel is open to question. As the UKMenCar4 study is entirely focused on adolescents, it may be better to emphasise the much higher age-specific carriage rates seen in that age group, rather than repeating the (often misinterpreted!) all-age population average of 10%.

 Notably, I believe the number of meningococcal whole-genome sequences (with corresponding MLST, rMLST cgMLST, capsular genogroup and BAST data analysis) that will be generated make this the largest study of it's kind to date.

 The commitment to also sequence other (non-pathogenic) *Neisseria *isolates is a welcome and valuable objective that may improve understanding of interactions with meningococcus within the oropharynx/nasopharynx.

 I look forward with great interest to seeing the data from this study in due course.

I confirm that I have read this submission and believe that I have an appropriate level of expertise to confirm that it is of an acceptable scientific standard.
